# HPV16 *E6/E*7 expression in circulating tumor cells in oropharyngeal squamous cell cancers: A pilot study

**DOI:** 10.1371/journal.pone.0215984

**Published:** 2019-05-09

**Authors:** Panagiota Economopoulou, George Koutsodontis, Margaritis Avgeris, Areti Strati, Christos Kroupis, Ioannis Pateras, Euthymios Kirodimos, Evangelos Giotakis, Ioannis Kotsantis, Pavlos Maragoudakis, Vassilis Gorgoulis, Andreas Scorilas, Evi Lianidou, Amanda Psyrri

**Affiliations:** 1 Section of Medical Oncology, Department of Internal Medicine, Faculty of Medicine, National and Kapodistrian University of Athens, Attikon University Hospital, Haidari, Athens, Greece; 2 Department of Biochemistry and Molecular Biology, Faculty of Biology, National and Kapodistrian University of Athens, Panepistimioupoli, Zografou, Athens, Greece; 3 Department of Chemistry, National and Kapodistrian University of Athens, Panepistimioupoli, Zografou, Athens, Greece; 4 Department of Clinical Biochemistry, School of Medicine, National and Kapodistrian University of Athens, Attikon University Hospital, Haidari, Athens, Greece; 5 Molecular Carcinogenesis Group, Department of Histology and Embryology, School of Medicine, National and Kapodistrian University of Athens, Goudi, Athens, Greece; 6 Department of Otolaryngology-Head and Neck Surgery, Hippokration General Hospital, University of Athens, Athens, Greece; 7 Department of Otorhinolaryngology, Facial Plastic and Reconstructive Surgery, Städtisches Klinikum Karlsruhe, Karlsruhe, Germany; 8 2nd Otolaryngology Department, Attikon University Hospital, Haidari, Athens, Greece; The Ohio State University, UNITED STATES

## Abstract

**Objectives:**

Human papillomavirus-related oropharyngeal squamous cell carcinoma (HPV+ OPSCC) is increasing in incidence. Although HPV+ OPSCC has favorable prognosis, 10 to 25% of HPV+ OPSCCs eventually recur. We sought to evaluate the feasibility of detection of HPV16 *E6/E7* expression in Circulating Tumor Cells (CTCs) and its utility as a prognostic tool in HPV16-associated OPSCC.

**Materials and methods:**

We developed a highly sensitive RT-qPCR assay for HPV mRNA expression in EpCAM(+) CTCs. In 22 patients with early stage and locally advanced OPSCC we evaluated HPV16 *E6/E7* expression in the EpCAM(+) CTC fraction at baseline and at the end of concurrent chemoradiotherapy. HPV status in pre-therapy formalin-fixed paraffin-embedded (FFPE) tumor biopsies was assessed by p16 immunohistochemistry and polymerase chain reaction (PCR) and double positives were subjected to Real-time qPCR assay for detection of HPV16, 18 and 31 types.

**Results:**

Fourteen of 22 OPSCC (63.6%) were HPV DNA+/p16+. Among HPV+/p16+ patients, 10 patients (71.4%) were HPV16 DNA+. HPV16 *E6/E7*(+) CTCs were detected in 3 of 10 patients (30%) at baseline and 4 of 9 patients (44.4%) at the end-of-treatment, all of which were p16+/HPV16 DNA+. Survival analysis showed a significantly higher risk for disease relapse (p = 0.001) and death (p = 0.005) in patients with HPV16 *E6/E7*(+) baseline CTCs.

**Conclusion:**

Detection of HPV *E6/E7*(+) CTCs might be a useful noninvasive test in liquid biopsy samples for determination of a clinically relevant HPV infection in HPV+ OPSCC. Combined interpretation of HPV *E6/E7*(+) CTCs with UICC staging data may lead to alteration of risk definition of patient subsets, with improved risk discrimination in early-stage disease.

## Introduction

Human papillomavirus (HPV) is a well-recognized etiologic factor in oropharyngeal squamous cell carcinoma (OPSCC), and it has been demonstrated that seropositivity for antibodies against HPV16 oncogenic proteins E6 /E7 is present in prediagnostic samples of patients with OPSCC many years before diagnosis [1]. HPV16 is the subtype most commonly implicated in the pathogenesis of OPSCC, with a prevalence over 90%, followed by HPV18 (3%) [2]. During the past few years, the global burden of the disease has steadily increased, and it has been predicted to surpass cervical cancer in some developed countries [3]. It is commonly accepted that HPV-associated oropharyngeal tumors (HPV+ OPSCC) comprise a distinct disease entity, showing a distinct clinical behavior and better survival outcomes [1]. However, approximately 10–25% of HPV+ OPSCCs have bad prognosis and eventually recur [4]; this population needs to be identified.

The capacity to measure tumor cells circulating through the vasculature (circulating tumor cells-CTCs) might provide evidence of the aggressiveness of a tumor prior to detection of identifiable metastases [5]. Studies have shown that CTCs in the peripheral blood could be a valuable tool for real-time monitoring of tumor status, predicting potential metastasis and recurrence, monitoring treatment efficacy and predicting survival of cancer patients [6]. In addition, carcinogenesis in HPV+ OPSCC is driven by sustained expression of viral *E6* and *E7* oncogenes. E6 inhibits p53, while E7 binds to retinoblastoma protein (pRb), promotes its degradation and the release of the E2F transcription factor, resulting in deregulation of the G1/S cell cycle check point and the activation of S-phase re-entry and viral replication [7]. Therefore, HPV-*E6/E7* oncogene expressing CTCs could be chosen as a unique biomarker for detection of viable tumor cells in the peripheral blood of OPSCC patients with transcriptionally active HPV infection.

Herein, we sought to assess whether CTCs expressing HPV16 *E6/E7* oncogenes (HPV16 *E6/E7*(+)) could be detected before initiation of treatment in OPSCC patients and correlate with treatment outcome. Thus, we aimed to evaluate HPV16 *E6/E7*(+) CTCs as a non-invasive prognostic tool in HPV16+ OPSCC.

## Patients and methods

### Study design

Twenty-two (n = 22) patients with early stage and locally advanced (LA) OPSCC were included in this analysis. Written informed consent was obtained from all patients before participating in the study. The present study was approved by the Medical Ethical Committee of Attikon University hospital (Athens, Greece) and complies with the principles laid down in the Declaration of Helsinki.

Pre-therapy tumor biopsies (FFPE tissue) were assessed for high risk (HR) HPV infection by p16 immunohistochemistry (IHC) and PCR (GP5^+^/6^+^ and MY systems), as well as for the detection of HPV16, 18 and 31 by qPCR. HPV DNA+/p16+ tumors were subjected to *E6/E7* oncogene expression evaluation by RT-qPCR.

OPSCC patients were treated with radical radiotherapy (early stage, n = 3) or cisplatin chemoradiotherapy +/- TPF (docetaxel, cisplatin, 5-fluorouracil) induction chemotherapy (IC) (locally advanced, n = 19). Nine (47.3%) of the nineteen patients with LA disease received IC. Patients were evaluated with head and neck MRI and chest CT scan +/- PET/CT scan at baseline and 3 months post radiotherapy completion. Liquid biopsy samples were obtained before treatment initiation. For 15 out of 22 patients, liquid biopsy samples were also available at the end of treatment. EpCAM(+) CTCs were immunomagnetically selected and subjected to HPV16 *E6/E7* oncogene expression analysis by RT-qPCR.

### Immunohistochemical (IHC) staining for p16

IHC was performed to determine *p16* expression using a p16 mouse monoclonal antibody (predilute, mtm-CINtech, E6H4) as previously described [8]. p16 was considered to be positive when defined as strong and diffuse nuclear and cytoplasmic staining in ≥70% of the tumor cells, which is the same scoring criteria used by Ang et al [8].

### DNA extraction from paraffin sections

Two 10 μm-paraffin sections from OPSCC specimens were used for DNA extraction by the QIAamp DNA FFPE Tissue kit (QIAGEN, Germany), according to manufacturer’s instructions. The extracted DNA was stored at -20°C until analysis.

### Detection of high-risk HPV DNA by PCR

High-risk HPV DNA detection was performed using the two most popular worldwide consensus PCR assays: the MY system [9] and the GP5^+^/6^+^ system [10] both amplifying regions of HPV *L1* gene. DNA integrity was assessed by PCR amplification of *β-globin* with PC04 and GH020 primers [9].

PCR reactions were performed in the GENEAmp PCR System 9600 (Applied Biosystems, USA). The 25 μl total reaction volume, contains 50–100 ng genomic DNA, 2.5 μl of 10X PCR buffer (w/o MgCl_2_), 3 μl of 10 mM dNTPs mix, 0.75 μl of 50 mM MgCl_2_, 1.75 μl of 10 μM forward primer, 1.75 μl of 10 μM reverse primer and 0.25 μl of 5 U/μl Platinum Taq DNA polymerase (Invitrogen, Life Technologies). Amplification for *β-globin* was performed at 94°C for 5 min followed by 36 cycles of 45 sec denaturation at 94°C, 45 sec annealing at 58°C and 45 sec elongation at 72°C. The last cycle was followed by a final extension of 5 min at 72°C. The thermal protocol for MY and GP5^+^/6^+^ systems consists of incubation at 94°C for 5 min followed by 40 cycles of 2 min denaturation at 94°C, 2 min annealing at 55°C for MY and 40°C for GP5^+^/6^+^ and 2 min elongation at 72°C. The last cycle was followed by a final extension of 5 min at 72°C. Appropriate controls including DEPC-H_2_O (blank), DNA-negative for HPV and DNA-positive for HPV16 from SiHa cervical carcinoma cells, were used. All necessary standard precautions were observed in order to avoid contamination through PCR carry-over. PCR products were analyzed in 1.5% w/v agarose gels.

### HPV RFLP typing

In case of HPV DNA+/p16+ samples, reactions were performed again in quadruplicate, mixed and their product was subjected to restriction fragment polymorphism (RFLP) analysis using the *BamHI*, *DdeI*, *HaeIII*, *HinfI*, *PstI* and *RsaI* restriction enzymes (New England Biolabs, USA). In precisely, 13 μl PCR product, 1.5 μl restriction buffer NEB 2 and 0.5 μl of each of the abovementioned restriction enzymes, in separate tubes, were incubated for 4h at 37°C. Analysis performed in 2% Nusieve 1:1 agarose gel as previously reported [11–12]. Assignment of an HPV type to a particular risk category was done according to Munoz et al [13].

### Real time qPCR for detection of HPV16, 18 and 31

Real-time qPCR assays were applied in HPV DNA+/p16+ FFPE samples in order to provide a more sensitive detection for HPV16, 18 and 31 types, most commonly found in OPSCC. The qPCR assays amplify a 93bp HPV16 *E6* region [14], a 185bp HPV18 *E1* region [15] and a 350bp HPV31 *E6* region [12].

The qPCR reactions were performed in the 7500 Real-Time PCR System (Applied Biosystems). The 10 μl reactions consisted of 50–100 ng genomic DNA, 5 μl of Kapa SYBR Fast Universal 2X qPCR Master Mix (Kapa Biosystems, Inc., Woburn, MA, USA) and 300 nM of each qPCR primer. The thermal protocol includes 3 min polymerase activation step at 95°C, followed by 40 cycles of denaturation at 95°C for 15 sec and the primer annealing and extension step at 60°C for 1 min. Duplicate reactions were performed for each tested sample. Melting curve analysis and agarose gel electrophoresis were performed following the amplification in order to distinguish the accumulation of the specific reaction products from non-specific ones or primer-dimers.

### Isolation of EpCAM^(+)^ CTCs

For the isolation of EpCAM(+) CTCs from peripheral blood (30mL) we followed our previously described protocols [16–18].

### RNA extraction

Total RNA was extracted from two 10 μm-paraffin sections using Nucleospin totalRNA FFPE XS (MACHEREY-NAGEL, Germany), with on-column rDNase digestion, according to manufacturer’s instructions. 1μg of the extracted RNA was further digested with DNAase I (Life technologies, USA) before cDNA synthesis. From the EpCAM(+) CTCs fraction, total RNA was isolated using the miRNeasy micro kit (QIAGEN, Germany), according to manufacturer’s instructions.

### cDNA synthesis

First-strand cDNA synthesis was carried out by the SuperScript First-Strand Synthesis System for RT-PCR (Life technologies, USA) according to manufacturer’s protocol, using 7μl of isolated total RNA as starting template.

### Detection of HPV16 E6/E7 expression in CTCs and paraffin embedded tissues

Expression analysis of HPV16 *E6/E7* oncogenes in CTCs and HPV16(+) tumor samples was performed by RT-qPCR, using specific qPCR primers for HPV16 *E6* and *β-actin* genes, amplifying a 109 bp HPV16 *E6*-specific and 105 bp *β-actin*-specific regions, previously described [12, 19].

The qPCR assay was performed in the 7500 Real-Time PCR System (Applied Biosystems). The 10 μl reaction mixture consists of Kapa SYBR Fast Universal 2X qPCR Master Mix (Kapa Biosystems), 300 nM of each qPCR primer and 2.0 μl cDNA, both for HPV16 *E6* and *β-actin* reactions. The thermal protocol consists of a 3 min polymerase activation step at 95°C, followed by 40 cycles of denaturation at 95°C for 15 sec and the primer annealing and extension step at 60°C for 1 min. Duplicate reactions were performed for each tested sample. Melting curve analysis and agarose gel electrophoresis were performed following the amplification in order to distinguish the accumulation of the specific reaction products from non-specific ones or primer-dimers.

### Statistical analysis

Patients’ recurrence and death were used as clinical endpoint events for progression-free survival (PFS) and overall survival (OS) of patients. PFS and OS were defined as the time from disease diagnosis to documented disease progression or patients’ death. Alive patients without documented events were censored at the time of the last disease evaluation. Co-primary objectives of the study were the associations between *E6/E7* expression at baseline and the end of treatment CTCs with PFS and OS intervals.

Chi-square test for categorical data used to assess the univariate differences of study parameters according to the gene expression. Survival analysis was performed by Kaplan-Meier curves, tested for significance by Mantel-Cox log-rank test. A level of p≤0.05 is considered statistically significant unless specified otherwise.

## Results

A total of 22 FFPE samples from patients with OPSCC were obtained and assessed for p16/HPV markers. Median follow-up was 31 months. Stage was determined according to American Joint Committee on Cancer (AJCC) 7^th^ edition TNM staging system. Baseline clinicopathological characteristics of patients are shown in Table 1.

**Table 1:** Clinicopathological features of OSCC patients.

Variable	No. of patients, n = 22	*p*-value^a^
**Tumor (T) classification**		
**T1**	**2** (9.1%)	0.45
**T2**	**7** (31.8%)	
**T3**	**4** (18.2%)	
**T4**	**9** (40.9%)	
**Nodal (N) classification**		
**N0**	**6** (27.3%)	1.00
**N+**	**16** (72.7%)	
**Stage**		
**II**	**3** (13.7%)	1.00
**III**	**1** (4.5%)	
**IVa**	**14** (63.6%)	
**IVb**	**4** (18.2%)	
**Grade**		
**1**	**1** (4.5%)	0.12
**2**	**11** (50.0%)	
**3**	**8** (36.4%)	
**unknown**	**2** (9.1%)	
**Tobacco consumption**		
**No**	**4** (18.2%)	0.61
**Ex Smoker**	**2** (9.1%)	
**Light Smoker**	**1** (4.5%)	
**Heavy Smoker**	**15** (68.2%)	
**Alcohol consumption**		
**No**	**8** (36.4%)	0.53
**Social**	**9** (40.9%)	
**Heavy**	**5** (22.7%)	
**Gender**		
**Male**	**16** (72.7%)	1.00
**Female**	**6** (27.3%)	

^a^ Chi-square test for the correlation of HPV16 *E6/E7* oncogene expression in baseline CTCs with HPV16(+) patients clinicopathological features

### p16 Immunohistochemistry

**Fig 1:**
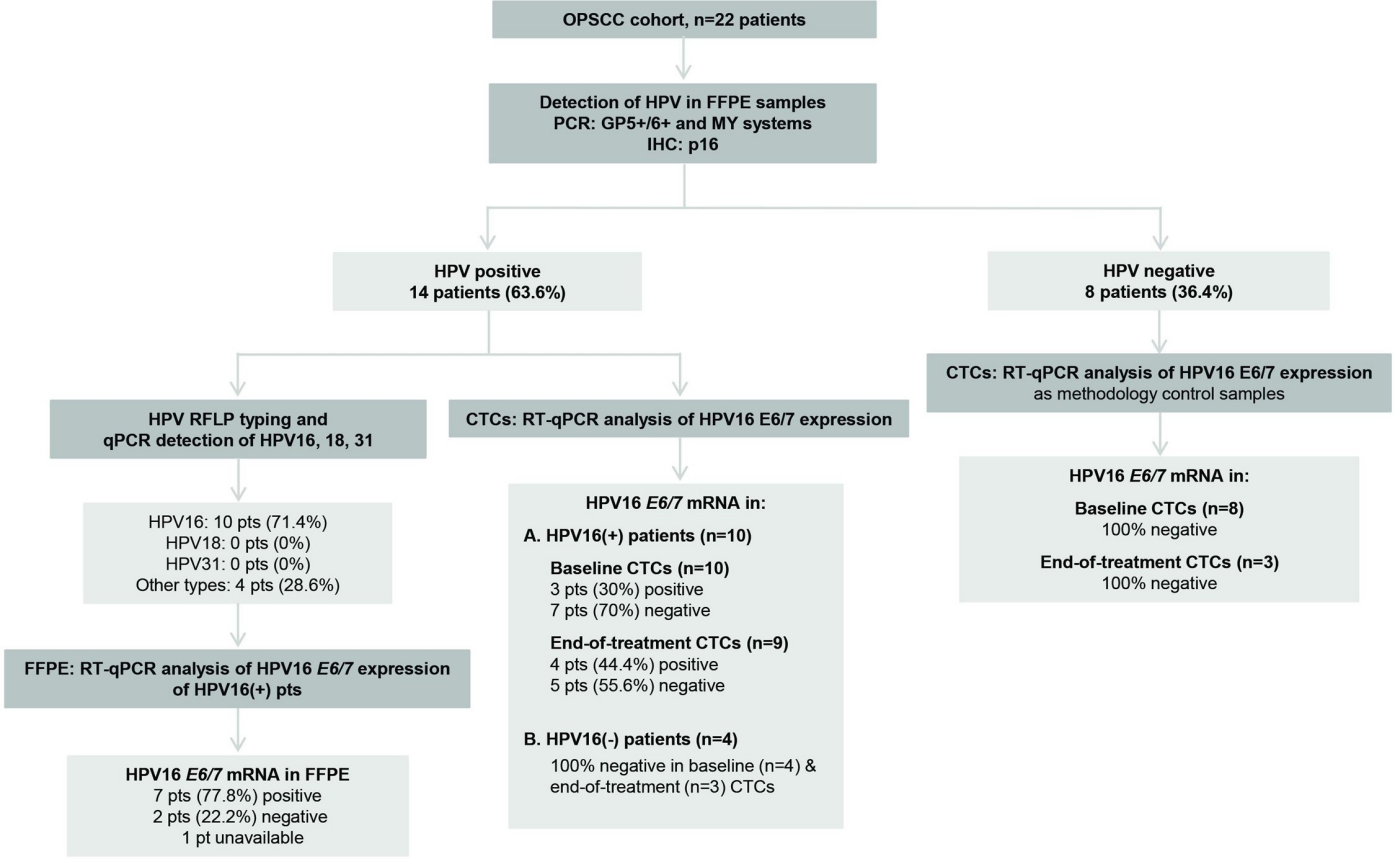
REMARK diagram of the study.

Twenty-two specimens were evaluable for p16 by immunohistochemistry. Fourteen of the twenty-two patients (63.6%) were positive for p16 (Fig 1).

#### Detection of high-risk HPV DNA by PCR

Twenty-two FFPE samples were evaluable for HPV DNA by PCR. All samples were subjected to histopathological evaluation, DNA quality control, and HPV DNA detection. HPV infection was detected in 14 of 22 OPSCC (63.6%) samples by PCR (GP5^+^/6^+^, MY systems) (Fig 1). Ten of 14 patients (71.4%) were HPV16+ by qPCR evaluation. Subsequently, HPV16 DNA+ tumors were subjected to HPV16 *E6/E7* oncogene expression analysis by RT-qPCR. Nine samples were evaluable for high-risk HPV16 mRNA analysis by RT-qPCR and HPV16 *E6/E7* expression was detected in 7 of them (77.8%) (Fig 1).

#### E6/E7 oncogene expression analysis in CTCs

HPV16 *E6/E7* oncogene expression analysis was performed in immunomagnetically positive selected CTCs isolated before treatment initiation, as well as the end of treatment for 15 patients of the cohort. HPV16 *E6/E7*(+) CTCs were detected in 3 of 10 HPV16+ patients (30%) at baseline, and in 4 of 9 HPV16+ patients (44.4%) at the end of treatment, all of which were p16+/HPV DNA+; however only 2 patients were HPV16 *E6/E7*(+) in both pre- and end-of-treatment CTCs. We found no HPV16 *E6/E7* mRNA expression in CTCs isolated from p16-/HPV DNA- or HPV16- patients. Clinicopathological characteristics of HPV16+ cases and the correlation with HPV16 E6/E7 positive CTCs are shown in Table 2.

**Table 2:** Clinicopathological characteristics of HPV16+ cases and correlation with HPV16 E6/E7 positive CTCs.

Case	Gender	Age at diagnosis	Stage	Tobacco	Alcohol	Treatment	CTCs at baseline	CTCs at the end of treatment
**1**	Μ	74	IVa	Heavy	Heavy	CRT	(+)	(+)
**2**	Μ	41	IVa	Νο	Νο	CRT	(-)	(-)
**3**	Μ	46	IVa	Heavy	Social	IC, CRT	(+)	(-)
**4**	F	55	IVa	Heavy	Social	IC, CRT	(-)	NA
**5**	Μ	42	IVb	Heavy	Social	CRT	(-)	(-)
**6**	Μ	45	II	Light	Social	CRT	(-)	(+)
**7**	F	44	IVa	Heavy	Social	CRT	(-)	(+)
**8**	M	76	IVa	Heavy	No	CRT	(-)	(-)
**9**	F	50	IVa	Heavy	Social	IC, CRT	(+)	(+)
**10**	Μ	52	IVa	Heavy	Heavy	CRT	(-)	(-)

**Abbreviations**: CRT = Chemoradiotherapy, F = Female, IC = Induction Chemotherapy, M = Male

#### Association with clinical outcome

**Fig 2:**
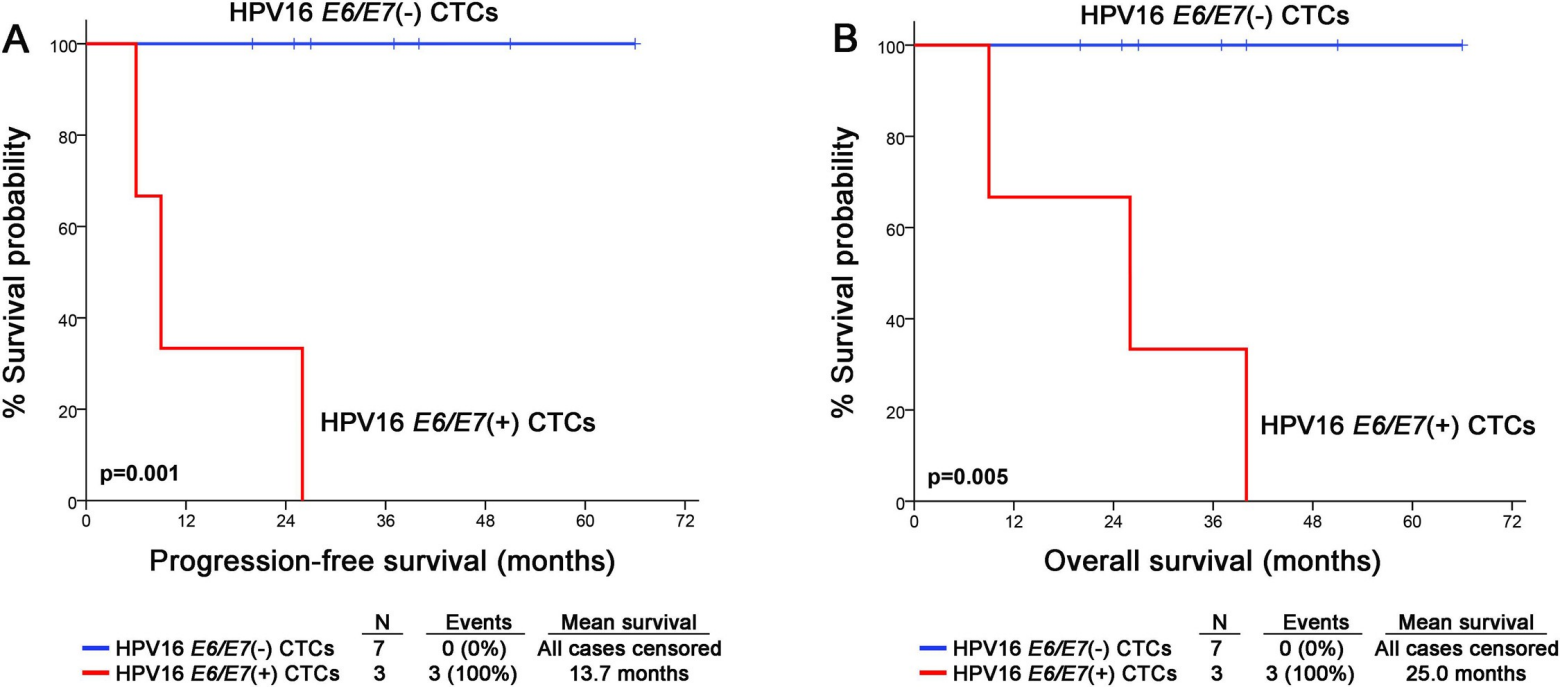
Survival analysis of OPSCC patients according to HPV16 *E6/E7* expression in CTCs. Kaplan-Meier survival curves for (A) the progression-free survival (PFS), and (B) the overall survival (OS) of patients. *P*-values were calculated by log-rank test.

Twenty-one patients were successfully followed-up, whereas one patient was excluded due to unclear monitoring data. During a median follow-up time (reverse Kaplan–Meier method) of 31 months (95% CI: 26.42–35.58) patients’ progression and death was detected in 7 (33.3%) patients. Among them, 4 patients were HPV16+ and 3 patients were HPV16-. Among HPV16+ cases, 3 patients had HPV16 E6/E7 positive CTCs at baseline and one had not. The mean PFS and OS was 45.41 months (95% CI: 33.15–57.68) and 44.39 months (95%CI: 31.86–56.92), respectively. The preplanned survival analysis of the HPV16+ patients showed a statistically significant higher risk for disease relapse (p = 0.001) and worse OS outcome (p = 0.005) in patients with HPV16 *E6/E7*(+) baseline CTCs. Kaplan-Meier curves are shown in Fig 2. Finally, Kaplan-Meier analysis did not show any significant correlation between *E6/E7* expression in CTCs isolated at the end of treatment and patients’ PFS (p = 0.596) or OS (p = 0.504) (S1 Fig). As expected, HPV+ OPSCC patients had improved prognosis in terms of PFS (p = 0.008) and OS (p = 0.049) compared to HPV- OPSCC. No statistically significant difference in prognosis was noted between HPV16 and HPV- other genotypes probably due to small numbers.

## Discussion

In the present report, we sought to evaluate whether HPV16 *E6/E7* oncogene expressing CTCs could be detected before and after treatment in a cohort of patients with OPSCC. Importantly, we show for the first time a significant association between HPV16 *E6/E7*(*+*) CTCs at baseline and both PFS (p = 0.001) and OS (p = 0.005). Although HPV+ OPSCC has favorable prognosis, 10% to 25% of HPV+ OPSCCs eventually recur. The identification of poor prognosis HPV16+ OPSCC has important treatment implications. The favorable prognosis of HPV+ OPSCC has fueled the development of deintensification strategies aiming to spare these patients the devastating consequences of aggressive treatment. HPV16 *E6/E7*(+) CTC detection could be used for better selection of deintensification candidates.

Although HPV16 is known to be responsible for more than 90% of HPV-related OPSCCs, in our cohort, only 71% of patients were found to be HPV16 positive. This discrepancy might be related to national differences, since Greece lacks official data registries regarding prevalence of HPV in head and neck cancer. Nevertheless, sample size is small to draw definite conclusions.

Several studies have emphasized the magnitude of molecular assays for CTC molecular characterization [16–18, 20]. Indeed, our group has demonstrated that real-time PCR-based methods performed in RNA or genomic DNA extracted from EpCAM(+) CTCs can provide useful information for the molecular characterization of CTCs. HPV *E6/E7* oncogene expression provides the most specific genetic marker distinguishing tumor cells bearing transcriptionally active HPV in peripheral blood [21]. In the present study, HPV16 *E6/E7*(*+*) CTCs were detected in 3 of 10 patients with HPV16(+) LA OPSCC (30%). Taking into account the paradigm of HPV-related cervical cancer, Tseng et al. found that the presence of HPV16 and HPV18 *E6* oncogene mRNA expression in the peripheral blood of patients with locally advanced disease was associated with bulky tumor volume and pelvic lymph node metastasis [22]. Furthermore, after a median follow-up of 22 months, patients with positive *E6* oncogene expression had a significantly higher risk of recurrence than those who were *E6* oncogene expression negative. Similarly, we found that patients with HPV16 *E6/E7*(*+*) CTCs before initiation of treatment had significantly higher risk for relapse and death. Thus, the clinical utility of HPV16 mRNA detection in CTCs isolated from patients with LA OPSCC deserves evaluation in large cohorts.

To date, this is the first study evaluating HPV16 *E6/E7* (+) CTCs as a prognostic tool in OPSCC patients. The majority of studies to date have evaluated the utility of circulating HPV DNA as a surveillance tool in patients with head and neck squamous cell carcinoma (HNSCC). A recent meta-analysis including 5 studies (n = 600 patients) reports a high pooled estimated specificity in detecting recurrence but an inferior pooled sensitivity [23]. Recent technical advances in detecting circulating DNA might improve the sensitivity [24]. Mazurek et al. based the diagnosis of HPV+ HNSCC only on detection of plasma HPV DNA using *E6/E7* PCR. The detection rate in that study was 14%. Interestingly, patients with more advanced disease had higher levels of circulating-free DNA [25].

Cao et al. detected HPV DNA in 65% of the pretreatment plasma samples from HPV+ OPSCC. Pre-treatment plasma HPV DNA copy number correlated significantly with nodal metabolic tumor volume (assessed on FDG-PET). Serial measurements in 14 patients showed rapid decline in HPV DNA that became undetectable at RT completion. Accordingly, post-treatment HPV DNA was elevated in 3 out of 4 patients with recurrent or residual disease prior to clinically and radiologically detected metastatic disease [26]. Dahlstrom et al. detected circulating HPV DNA in pre-treatment serum and found that the presence of circulating HPV DNA was associated with higher N category and overall stage. Although patients with HPV+ tumors with detectable pre-treatment levels of circulating HPV DNA had worse PFS, this difference did not reach statistical significance [27]. Similarly, Lee et al. recently demonstrated that detection of HPV DNA in sequential samples through and after chemo-radiotherapy predicted response and residual disease at the primary site and in cervical lymph nodes in patients with HPV + LA disease [28].

One important application of our results is the identification of OPSCC patients with poor prognosis. Current clinical trials address separately HPV+ and HPV- OPSCC and focus on treatment deintensification. If validated, the test described in this paper could be easily incorporated into routine clinical practice as a prognostic tool for therapy selection. Importantly, in this small cohort there was no association between high tumor burden and the presence of HPV16 *E6/E7*(+) CTCs at baseline indicating that the latter might be an independent prognostic marker in HPV+ OPSCC. Due to small sample size we did not find a significant correlation between clinical outcome and HPV16 *E6/E7*(+) CTCs at the end of treatment; a prospective study evaluating this question in a large number of patients is needed.

A major limitation of our study is that it is a single institution small cohort and our results need to be validated in large cohorts with longer follow-up. In addition, EpCAM-based isolation methods may miss CTCs bearing mesenchymal rather than epithelial phenotype, a phenomenon that is not uncommon in HNSCC.

In conclusion, we sought to assess the feasibility of detection of HPV *E6/E7*(+) CTCs in OPSCC. We demonstrated a significant association between HPV16 *E6/E7* mRNA detection and clinical outcome in this pilot cohort. Combined interpretation of HPV *E6/E7*(+) CTCs with UICC staging data may lead to alteration of risk definition of patient subsets, with improved risk discrimination in early-stage disease. Validation studies are awaited.

## Supporting information

S1 Fig Survival analysis of OPSCC patients according to HPV 16 E6/E7 expression in CTCs at the end of treatment. Kaplan-Meier survival curves for (a) Progression Free Survival (PFS) and (b) Overall Survival (OS) of patients. P values were calculated by long-rank test.
(TIF)
