# Supplementary figures and images for: HPV16 E6/E7 expression in circulating tumor cells in oropharyngeal squamous cell cancers: A pilot study
Source: PLoS One. 2019 May 9;14(5):e0215984. doi: 10.1371/journal.pone.0215984 (PMC6508656; doi:10.1371/journal.pone.0215984)

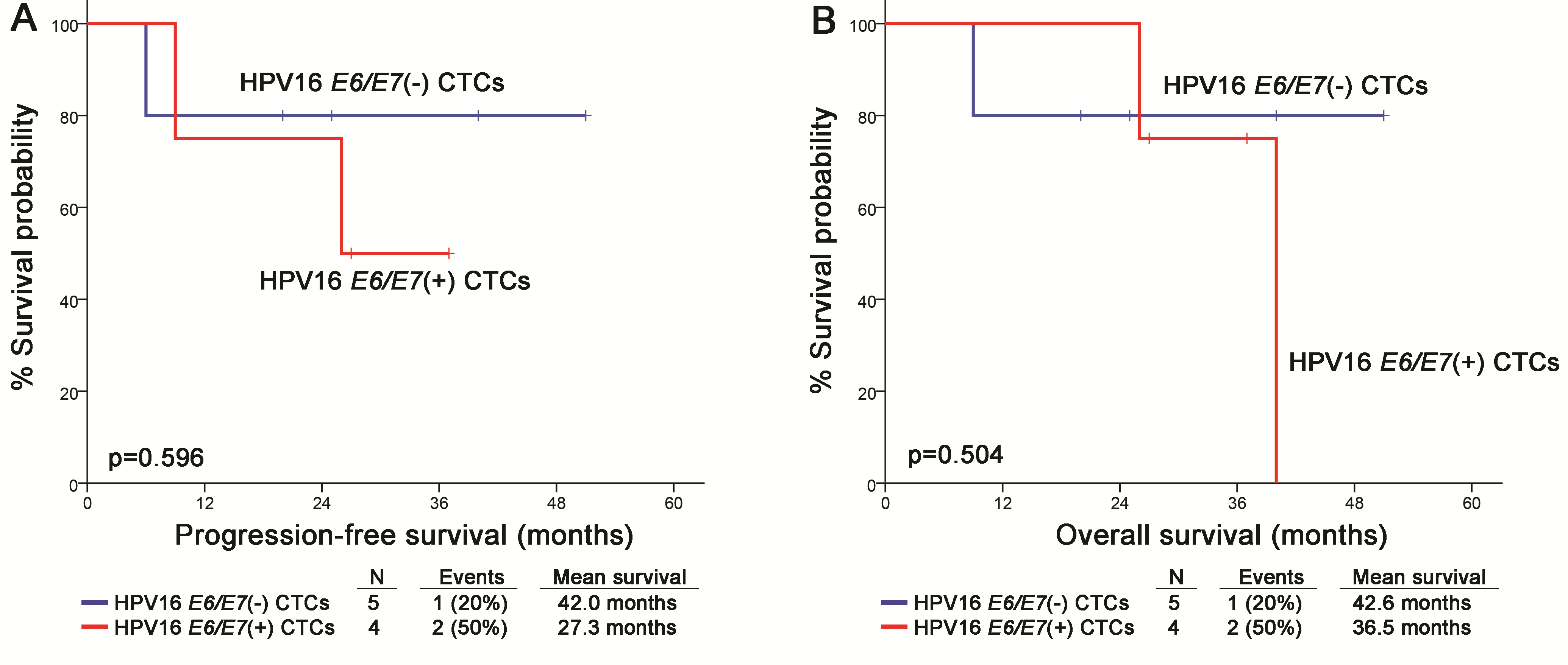

Supplement: S1 Fig — Kaplan-Meier survival curves for (a) Progression Free Survival (PFS) and (b) Overall Survival (OS) of patients. P values were calculated by long-rank test. (TIF) [file pone.0215984.s001.tif]
